# Metagenomic detection of protozoan parasites on leafy greens aided by a rapid and efficient DNA extraction protocol

**DOI:** 10.3389/fmicb.2025.1566579

**Published:** 2025-03-14

**Authors:** Sohail Naushad, Ruimin Gao, Marc-Olivier Duceppe, Andree Ann Dupras, Sarah J. Reiling, Harriet Merks, Brent Dixon, Dele Ogunremi

**Affiliations:** ^1^Ottawa Laboratory Fallowfield, Canadian Food Inspection Agency, Ottawa, ON, Canada; ^2^Bureau of Microbial Hazards, Food and Nutrition Directorate, Health Canada, Ottawa, ON, Canada

**Keywords:** metagenomics, MinION, diagnostics, *Cryptosporidium*, *Giardia*, *Toxoplasma*

## Abstract

**Introduction:**

Infections with protozoan parasites associated with the consumption of fresh produce is an on-going issue in developed countries but mitigating the risk is hampered by the lack of adequate methods for their detection and identification.

**Materials and methods:**

We developed a metagenomic next-generation sequencing (mNGS) assay using a MinION sequencer for the identification of parasites in intentionally contaminated lettuce to achieve a more accurate and rapid method than the traditional molecular and microscopy methods commonly used for regulatory purposes. Lettuce (25 g) was spiked with varying numbers of *Cryptosporidium parvum* oocysts, and microbes washed from the surface of the lettuce were lysed using the OmniLyse device. DNA was then extracted by acetate precipitation, followed by whole genome amplification. The amplified DNA was sequenced by nanopore technology and validated with the Ion Gene Studio S5, and the generated fastq files raw reads were uploaded to the CosmosID webserver for the bioinformatic identification of microbes in the metagenome. To demonstrate the ability of the procedure to distinguish other common food and waterborne protozoan parasites, lettuce was also spiked with *C. hominis, C. muris, Giardia duodenalis* and *Toxoplasma gondii* individually or together.

**Results:**

The efficient lysis of oocysts and cysts was a prerequisite for the sensitive detection of parasite DNA and was rapidly achieved within 3 min. Amplification of extracted DNA led to the generation of 0.16–8.25 μg of DNA (median = 4.10 μg), sufficient to perform mNGS. Nanopore sequencing followed by bioinformatic analysis led to the consistent identification of as few as 100 oocysts of *C. parvum* in 25 g of fresh lettuce. Similar results were obtained using the Ion S5 sequencing platform. The assay proved useful for the simultaneous detection of *C. parvum, C. hominis, C. muris, G. duodenalis* and *T. gondii*.

**Discussion:**

Our metagenomic procedure led to the identification of *C. parvum* present on lettuce at low numbers and successfully identified and differentiated other protozoa either of the same genus or of different genera. This novel mNGS assay has the potential for application as a single universal test for the detection of foodborne parasites, and the subtyping of parasites for foodborne outbreak investigations and surveillance studies.

## 1 Introduction

Foodborne parasitic organisms are the causative agents of numerous devastating and prevalent infections worldwide (Robertson et al., 2018; Torgerson et al., 2015). While person-to person and waterborne transmissions are very common for parasites such as *Cryptosporidium* spp. and *Giardia duodenalis*, transmission through the consumption of contaminated foods is an emerging public health issue in many parts of the world (Dixon et al., 2011, 2013). Fresh produce, particularly vegetables, fruits and herbs, is increasingly a source of foodborne illnesses (Dixon et al., 2013; Vizon et al., 2019), and tested in surveillance studies in different parts of the world (Iqbal et al., 2015; Thivierge et al., 2016). Contamination may occur at different stages in the food chain: pre-harvest, post-harvest or immediately before consumption (Gamble, 2015; Robertson, 2018).

*Cryptosporidium* and *Giardia* are protozoan parasites responsible for diseases known as cryptosporidiosis and giardiasis, respectively. These diseases are among the most common intestinal diseases in humans and animals and are considered serious global public and veterinary health concerns (Robertson et al., 2018; Squire and Ryan, 2017). Numerous studies have reported the occurrence and distribution of *Cryptosporidium* spp. and *G. duodenalis* on fresh leafy vegetables, soil, and water (Budu-Amoako et al., 2011; Checkley et al., 2015; Lalonde and Gajadhar, 2016; Prystajecky et al., 2014; Ryan et al., 2019).

Many available molecular methods such as loop-mediated isothermal amplification (LAMP), PCR, nested PCR, and real time qPCR are in use for parasite identification (Ricciardi and Ndao, 2015; Pomari et al., 2019; Gomez et al., 2019; Wong et al., 2018). However, microscopic identification is considered the gold standard (Ndao, 2009). These approaches have contributed to advances in the detection of parasitic infections but all have their important limitations. For example, molecular methods rely on *a priori* knowledge of the relevant attributes of the organism(s) of interest, and can target only one or a few organisms at a time (Kelly et al., 2019). Additionally, molecular methods such as PCR are limited by the need to extract good quality DNA from the parasites present in the sample to avoid polymerase enzyme inhibition, which is often difficult due to the robustness of oocyst/cyst walls. Many commercial kits and methods are available for the extraction of DNA from bacteria or viruses, but are inefficient for parasites. Traditionally, oocysts/cysts are lysed using repeated rounds of quick freeze-thaw cycles in liquid nitrogen (Ricciardi and Ndao, 2015). Although this method has resulted in the recovery of detectable amounts of DNA, it is time consuming and cannot be easily adapted for field testing (Momčilović et al., 2019). Heating of oocysts/cysts at 100°C for 10–15 min has also been tested for the recovery of parasite DNA. This method aids in the lysis of the oocyst/cyst wall, but also interferes with the integrity of double-stranded DNA (Hawash, 2014). Additionally, many of these methods fail to yield sufficiently high quality DNA required for next-generation DNA sequencing (NGS) (Ricciardi and Ndao, 2015). NGS has shown tremendous progress over the past decade, transforming genomic analysis and opening up new opportunities for the development of novel, improved and culture-independent diagnostic methods for bacterial and viral infections (Wylezich et al., 2019; Ogunremi et al., 2020). Currently, there is no NGS-based method that can simultaneously identify and differentiate various parasites. NGS technology can be adapted into a single test to address diagnostic needs which previously required several, individual tests (Chiang and Dekker, 2020; Pallen, 2014).

The availability of novel DNA sequencing platforms such as the MinION (Oxford Nanopore Technologies) has opened up the opportunities to sequence metagenomes and produce many gigabases of sequence data within a few hours. Metagenomic sequence data enables the characterization of organisms at the genus, species, and even genotype levels (Forbes et al., 2017; Mechan-Llontop et al., 2020; Ogunremi et al., 2020). Additionally, metagenomic approaches, compared to the targeted approaches described above (e.g., PCR), allow for comprehensive testing because they require no prior knowledge of the potential pathogens present in a sample. However, the availability of an accurate and highly curated database is a prerequisite for reliable metagenomic analyses.

The objectives of this study were to (1) develop a rapid and efficient method for the extraction of sequencing quality DNA from parasite oocysts/cysts; (2) amplify extracted DNA to quantities required for various NGS sequencing technologies; (3) develop a metagenomic sequencing and identification workflow for *Cryptosporidium* spp., *G. duodenalis* and *Toxoplasma gondii* using MinION sequencing technology; (4) validate MinION sequencing using an alternate sequence chemistry, i.e., Ion S5 system sequencing; and (5) establish the limit of parasite detection.

## 2 Materials and methods

### 2.1 Preparation of parasite suspensions and spiking of lettuce

Live highly purified suspensions of *C. parvum, C. hominis*, and *C. muris* oocysts and *G. duodenalis* cysts were purchased from Waterborne Inc. (New Orleans, LA). *Toxoplasma gondii* oocysts were generously provided by Dr. J. P. Dubey, United States Department of Agriculture. Counts of concentrated parasite suspensions in phosphate buffered saline, PBS (pH 7.2) were estimated by light microscopy at 100 × magnification (Olympus BX51, Tokyo, Japan) with the aid of a Neubauer hemocytometer counting chamber. The count, integrity and intactness of the oocysts/cysts in the diluted suspensions were confirmed before spiking lettuce by checking a replicate sample that had been concentrated to ~10 μl by centrifugation and placed on a glass slide without a coverslip allowing the droplet to be scanned under the microscope at 100×. Individual romaine lettuce (*Lactuca sativa* L. var. *longifolia*) leaves (~25 g, as recommended by international food safety regulatory authorities, for example see The Compendium of Analytical Methods—Canada.ca), were placed flat in a sterile plastic container in a Biological Safety Cabinet with the fan turned on. Replicate lettuce samples (n) were spiked with 1 ml each containing the following number of *C. parvum* oocysts: 1 (*n* = 4), 2 (*n* = 2), 5 (*n* = 2), 10 (*n* = 5), 25 (*n* = 2), 100 (*n* = 2), 250 (*n* = 1), 500 (*n* = 3), 1,000 (*n* = 3), 10,000 (*n* = 2), and 100,000 (*n* = 3), added dropwise over the entire surface of the leaves. At the same time, 25 g lettuce leaves spiked with 1 ml of PBS were used as a negative control in all experiments. Spiked leaves were left to air dry for at least 15 min, after which leaves having completely absorbed the spiking fluid were placed in individual stomacher bags (Seward, Worthing, England) containing 40 ml of buffered peptone water supplemented with 0.1% Tween. Oocysts and other debris were dissociated from the lettuce surface in a stomacher at 115 rpm for 1 min, and then fluid from each bag was passed through a custom-made 35 μm filter with a vacuum pressure to remove particulate matter, including plant material. The filtrate, including oocysts, was pelleted by centrifugation at 15,000x *g* for 60 min at 4°C (Sorvall RC 5B, DuPont Company, Wilmington, DE) and the supernatant was discarded. To establish whether the method could differentiate among various parasite species, separate lettuce leaves were spiked with 1 ml of PBS solution each containing *C. hominis* (1,000–100,000 oocysts), or *C. muris* (1,000–100,000 oocysts) or *G. duodenalis* (100–100,000 cysts). To evaluate the performance of the assay in identifying and differentiating among various parasite species, a lettuce leaf was spiked with a mixture of four parasites (6 × 10^4^ oocysts/cysts each of *C. parvum* or *T. gondii*, 3 × 10^4^ of *C. hominis* and 5 × 10^3^ of *G. duodenalis*). The recovery of the parasites from spiked lettuce was carried out as described above.

### 2.2 Lysis of oocysts/cysts

The pellet obtained after the filtration and centrifugation of the stomacher fluid was suspended in 500 μl PBS (pH 7.2), and the parasites present were lysed using the OmniLyse^®^ Lysis Kit (Claremont BioSolutions, Upland, CA). The OmniLyse lysis kit is operated by a battery pack and utilizes a bead beating method to quickly disrupt hard cells, including cysts, without the use of any harsh chemicals, water baths, or a centrifuge. The OmniLyse device contains a 3 ml disposable syringe and a mixing chamber that has a small motor equipped with a “precision-cut impellor” along with the zirconia/silica beads that operates at ≥ 30,000 rpm, generating a high shear force that disrupted thick cell walls. The mixing chamber was pre-washed with 1 ml of PBS by passing the buffer back and forth 6–8 times for 1 min, with the power on. After 1 min, the unit was turned off and the PBS was discarded. Once this pre-wash process was completed, the syringe was used to aspirate 500 μl of resuspended pellet into the OmniLyse^®^ cartridge before the unit was turned back on. The remainder of the sample was then passed back and forth in the chamber ~15 times over a 3 min period after which the power was turned off and the lysate collected in a clean 1.5 ml microcentrifuge tube.

### 2.3 DNA precipitation and enrichment

DNA precipitation was carried out by adding 50 μl of sodium acetate (3M, pH 5.2) to 500 μl of lysate to bring the final salt concentration to 0.3M. Thereafter, 550 μl (1 volume) of isopropanol kept at room temperature was added to the lysate-salt solution. Samples were then centrifuged at 15,000 × g for 30 min at 4°C to pellet the DNA. To remove the co-precipitated salt from the DNA, pellets were washed twice with 70% ethanol at 15,000 × g for 15 min at 4°C, air-dried for 5 min at room temperature and re-suspended in 9 μl of ultrapure distilled water. To ensure that sufficient DNA quantities were available for whole genome sequencing, whole genome amplification was carried out using the GenomePlex^®^ WGA4 kit (Sigma Aldrich, Oakville, ON) as described (Ogunremi et al., 2020). Briefly, 1 μl of lysis and fragmentation buffer (2 μl proteinase K solution added to 32 μl of 10 × single cell lysis and fragmentation buffer) was added to 9 μl DNA preparation followed by incubation for 1 h at 50°C and heating at 99°C for 4 min. A library preparation step was performed as part of the whole genome amplification procedure and involved adding 2 μl of library preparation buffer to 1 μl of library stabilization solution followed by incubation in a thermal cycler at 95°C for 2 min. The tube was placed on ice after this incubation step and 1 μl of library preparation enzyme was added followed by incubation at 16, 24, and 37°C for 20 min each, and finally at 75°C for 5 min. For the amplification of the library, 7.5μl of amplification master mix and 5 μl WGA DNA Polymerase was added, and the final volume was brought to 77 μl by adding 48.5 μl distilled water to each reaction. The amplification step was carried out in a thermocycler with an initial denaturation at 95°C for 3 min followed by 25 cycles of annealing at 94°C for 30 s and an extension at 65°C for 5 min. Amplified DNA was purified using the GeneJet PCR purification kit (Thermo Scientific, Burlington, ON) following the manufacturer's instructions. The quality and quantity of the purified DNA was determined with a spectrophotometer (DU 730 Beckman Coulter, Mississauga, ON), and Qubit 2.0 (Life Technologies, Carlsbad, CA), respectively.

### 2.4 Library preparation and MinION sequencing

Library preparation for MinION sequencing was performed using a 1D ligation sequencing kit (SQK-LSK109) and native barcoding kit (EXP-NBD104; Oxford Nanopore Technologies, Oxford, England). In general, the manufacturer's protocol was followed but modified to accommodate the 600 bp average DNA fragment size obtained at the end of the genome amplification step, which was performed before preparing the DNA library for sequencing. The first modification was the use of 250 fmol DNA as calculated using NEBioCalculator v1.10.0 (https://nebiocalculator.neb.com/#!/dsdnaamt) instead of the recommended 1 μg, to ensure an optimal molar ratio between the ends of the amplified DNA fragments and the native barcodes. The second modification was the extension time for DNA repair and dA-tailing to 15 min at 20°C and 15 min at 65°C, respectively, as opposed to the 5 min recommended for each procedure. Repair and dA-tailing were achieved by mixing 3.5 μl of NEBNext FFPE DNA Repair Buffer, 2 μl of NEBNext FFPE DNA Repair Mix (New England Biolab, M6630), 7 μl NEBNext Ultra II End Prep Reaction Buffer and 3 μl NEBNext Ultra II End Prep Enzyme Mix (New England Biolab, E7546). Individual barcodes were ligated to dA-tailed DNA using 4 μl (as opposed to the recommended 2.5 μl) of barcode and 25 μl NEB Blunt/TA Master Mix for 10 min at room temperature. Barcoded samples were quantified using the Qubit dsBR DNA assay (Life Technologies, Carlsbad, CA) and pooled in equimolar concentrations. A pre-sequencing library (PSL) was prepared by adding 5 μl of Adapter Mix II (SQK-LSK109) to 65 μl of pooled barcoded-DNA and ligated for 10 min at room temperature with 20 μl of NEBNext Quick Ligation Reaction Buffer (5 × ) and 10 μl Quick T4 DNA Ligase (New England Biolab, E6056). The final PSL was eluted in 15 μl of EB buffer (SQK-LSK109) and quantified using the Qubit assay. Each flow cell (FLO-MIN106) was primed according to the manufacturer's guidelines before loading with 75 μl mix containing 12 μl of PSL (50 fmol), 37.5 μl SQB buffer (SQK-LSK109), and 25.5 μl loading beads (LB) (SQK-LSK109). The loaded flow cell was mounted on the MinION chassis, and sequencing was guided by the MinKNOW software for up to 48 h and data managed with the MinIT device (Oxford Nanopore).

### 2.5 Basecalling, demultiplexing, quality control, and bioinformatic analyses of minion data

Raw fast5 files generated at the end of MinION sequencing runs were transferred to a separate high power Linux computer, and the sequence reads were trimmed of adaptors, base-called using the “High Accuracy” basecalling algorithm in Guppy (v3.1.5) and demultiplexed resulting in the sorting of reads into the pass and fail bins, and according to the barcodes. The quality of the sequencing run was assessed with an in-house built program; nanoQC v0.1 (https://github.com/duceppemo/nanoQC). A minimum of 80,000 fastq reads was deemed necessary to continue the analysis. Parasites were identified by uploading fastq reads to CosmosID webportal (CosmosID GENIUS^®^) (https://app.cosmosid.com/samples). CosmosID shotgun metagenomics analysis involved the screening of hundreds of millions of short reads against a highly curated database of fixed length *k-mer* fingerprints or biomarkers to disambiguate sequence reads into discrete microorganisms (http://www.cosmosid.com). Description of the pipeline and the proprietary GenBook^®^ database were previously provided (Ogunremi et al., 2020). To establish how quickly parasites could be detected, we performed simulated time series experiments on MinION data by exploiting the time stamp on each sequence read generated by the MinION sequencer. For these analyses, we tested samples spiked with 1,000 oocysts of *C. parvum* per 25 g of lettuce. We sorted pass MinION reads and arranged them according to the time of sequencing using nanoTimeSort v0.1 (https://github.com/duceppemo/nanoTimeSort). We developed the script to simulate a time series study by segregating MinION output into 30 min of sequencing reads with sequential additions, i.e., the first folder contains the first set of reads acquired over a 30 min time span and the second folder contains the cumulative sequence reads from the first 30 min and the following 30 min reads. The process continues until the end of the run at about 48 h.

### 2.6 Ion GeneStudio S5 sequencing

To validate the results of the MinION sequencing procedure, Ion S5 sequencing DNA libraries were generated using 1 μg of the WGA4-amplified DNA by means of an Ion Xpress™ plus gDNA fragment library preparation kit (ThermoFisher, Burlington, ON) and sequenced on the Ion GeneStudio S5 sequencer as previously described (Ogunremi et al., 2020). The average size of the DNA fragments present in the library was assessed using an Agilent High Sensitivity kit on an Agilent Bioanalyzer (Agilent, Santa Clara, CA) and confirmed to be ~400 bp, as recommended for the Ion S5 platform by the manufacturer. Specifically, each prepared library was quantified using an Ion library TaqMan quantification kit (Cat. No. 4468802) and was diluted to 45 pM for the subsequent automated template preparation, enrichment and Ion 530 chip loading on Ion Chef (ThermoFisher). Given the high capacity of the 530 chip, two libraries were loaded per chip and sequenced for 4.5 h. At the end of the run, fastq reads were downloaded from the Ion Torrent server and analyzed using CosmosID software, as described above.

## 3 Results

### 3.1 Extraction and enrichment of DNA from oocysts/cysts

We evaluated the capability of the OmniLyse device to quickly disrupt oocysts/cysts and liberate DNA suitable for sequencing with NGS technologies. The yield and purity of each sample was assessed, and the total DNA yield of material containing different parasite spiking numbers ranged from 0.01 to 0.43 μg (median = 0.06 μg; Supplementary Table S1). Because NGS technologies such as MinION and Ion S5 sequencing require high quantities of starting DNA, we amplified the DNA using the WGA4 whole genome amplification method. WGA4 amplification produced 0.16–8.25 μg total DNA (median = 4.10 μg; Supplementary Table S1), which was sufficient for sequencing with the MinION and Ion S5 sequencers. The purity of the amplified DNA was assessed by determining DNA absorbance A_260_/A_280_ ratios, which ranged from 1.14 to 1.75 and reflected the presence of impurities (Supplementary Table S1). Each DNA preparation was cleaned with GeneJet PCR clean up kit and the A_260_/A_280_ ratios improved to 1.79–2.09 as required for sequencing according to the manufacturer's instructions (Supplementary Table S1).

### 3.2 MinION and Ion S5 metagenomic sequencing and the detection of parasites from the spiked and non-spiked sequencing data

The MinION sequence reads were base called and demultiplexed and each sample had 0.08M−13.48M fastq reads (Supplementary Table S1). The analysis to detect parasites from the spiked and non-spiked lettuce was carried out by uploading fastq reads to the CosmosID website. In the sequencing reads obtained from spiked lettuce, *C. parvum* was consistently identified in all samples spiked with 100 or more *C. parvum* oocysts (Table 1). Remarkably, CosmosID analysis detected only *C. parvum* in the sample, with no hits observed for any other parasite. In all spiked samples, CosmosID analysis also provided results regarding the presence of various bacterial genera that make up the normal surface biota of the lettuce. The most prominent bacterial genera found on the surface of the fresh romaine lettuce were: *Pantoea, Pseudomonas, Acinetobacter, Flavobacterium, Methylbacterium, Sphingomonas, Pedobacter, Chryseobacterium, Xanthomonas, Bacillus, Achromobacter, Pseudoxathomonas, Enterobacter, Pandoraea, Dyella*, and *Citrobacter* in agreement with previous observations using the Ion Torrent Personal Genome Machine by Ogunremi et al. (2020). Non-spiked samples were negative for *C. parvum* and other parasites (*n* = 12; Table 1).

**Table 1:** Detection of protozoan parasites by metagenomic nanopore sequencing following intentional contamination of lettuce.

	**No of oocysts/ cysts**	**Replicates (*n*)**	**Number testing positive**
*Cryptosporidium parvum*	1	4	0
	2	2	1
	5	2	1
	10	5	5
	25	2	1
	100	2	2
	250	1	1
	500	3	3
	1,000	3	3
	10,000	2	2
	100,000	3	3
*Cryptosporidium muris*	1,000	2	2
	5,000	2	2
	100,000	1	1
*Cryptosporidium hominis*	1,000	1	1
	5,000	2	2
	100,000	1	1
*Toxoplasma gondii*	100,000	1	1
*Giardia duodenalis*	100	1	1
	1,000	1	1
	5,000	1	1
	100,000	2	2
Phosphate buffered saline	0	12	0

Lettuce (25 g) was spiked with indicated number of parasite oocysts/cysts suspended in Phosphate Buffered Saline (PBS) in replicates of n, and DNA extracted as outlined under Materials and Methods. Negative controls were exposed to PBS only. A metagenomic DNA library was prepared for each sample and followed by nanopore sequencing using a MK1C sequencer (Oxford Nanopore Technologies, Oxford United Kingdom). The presence of parasite sequences was determined using the proprietary, web-based bioinformatic service provided by CosmosID (Germantown, MD, USA).

In order to evaluate the specificity of detection, we performed experiments involving the spiking of lettuce with the various protozoan oocysts/cysts: *C. parvum, C. hominis, T. gondii* and *G. duodenalis*. From the subsequent CosmosID analyses, we were able to specifically identify and distinguish all four parasite species (Supplementary Figure S1). To further validate our procedure, sequencing reads obtained from non-spiked lettuce were also analyzed using CosmosID. Parasites were not detected in any of the control, non-spiked lettuce samples (*n* = 12). However, a similar bacterial composition as observed in spiked lettuce samples was seen on non-spiked lettuce, confirming their existence as normal microbiota. To determine the speed of parasite detection, sequencing reads that were sorted according to the time of sequencing in 30 min intervals were analyzed using CosmosID procedure and the earliest time of detecting parasites was 1.5 h after the start of the sequencing run (Supplementary Table S2). A subset of samples spiked with *C. parvum* (1, 2, 5, 25, 10,000, and 100,000) was sequenced with Ion S5 sequencing in an attempt to validate the MinION sequencing. In the same manner as the MinION data analysis, Ion S5 reads were also uploaded to the CosmosID website and the presence or absence of parasites was confirmed. In the CosmosID analyses of Ion S5 data, respective parasites were consistently detected at a spike level of ≥10 oocysts in each contaminated lettuce sample (Supplementary Table S3).

## 4 Discussion

We have developed a rapid and efficient procedure for recovering DNA of high quantity and purity from parasites that can be used for NGS technologies to ensure reliable and reproducible results. Current molecular methods for parasite detection are hindered by the difficulty in recovering DNA from oocysts/cysts, which are usually present in low abundance compared to bacterial communities (Momčilović et al., 2019; Pallen, 2014). Many methods are available for parasite oocyst/cyst wall disruption, including physical approaches (e.g., particle-based disruption and sonication), physiochemical methods (e.g., use of detergents, enzymes, temperature, etc.) and commercial kits, but all have limitations in recovering DNA (Ndao, 2009; Ricciardi and Ndao, 2015). In a comparative study, the authors determined that the most successful method for extracting DNA from oocysts involved multiple (5–15) cycles of freezing (liquid nitrogen) and thawing (65°C) in lysis buffer (Nair et al., 2014). Although efficient, the method is laborious and time consuming for oocyst lysis. Additionally, because of the logistical requirements, such methods cannot be adopted for field testing or in laboratory settings with minimal equipment and infrastructure. Commercial kits such as QIAamp DNA stool mini (Qiagen), SpeedTools DNA extraction (Biotools), DNAExtract-VK (Vacunek), PowerFecal DNA isolation (MoBio), and Wizard magnetic DNA purification system (Promega Corporation) have also been used for parasite DNA extraction (Hart et al., 2015; Nair et al., 2014) and while offering good performance, cost-effectiveness or ease of use, they often fail to recover sufficient DNA when applied to samples with low parasite count. Extensive modification of protocols are often required including repeated freeze-thaw cycles in liquid nitrogen, heating the oocysts/cysts at 100°C for 10–15 min or treating samples with proteinase K for 8–12 h (Hart et al., 2015; Nair et al., 2014).

We used the OmniLyse device which disrupts the oocysts/cysts wall within 3 min. The performance of the OmniLyse kit in the disruption of hard-to-break bacterial cell walls was shown to have similar or superior results to many commercial kits, bead beating and sonication methods (Vandeventer et al., 2011). Additionally, bead beating methods such as Mini-Bead Beater and sonication methods such as VibraCell Ultrasonic system are larger, heavier, and require bench-top devices and significant power resources to operate (Vandeventer et al., 2011), making them less feasible for use in a field setting. The quantity of DNA recovered after OmniLyse is not sufficient for NGS technologies, such as MinION and Ion S5 sequencing, where 1 μg DNA is recommended for optimal sequencing results. Therefore, a critical step for NGS sequencing is to generate sufficient quality and quantity of DNA for a sequencing library by whole genome amplification. In our experiments, total DNA recovery using the OmniLyse procedure ranged from 0.01 to 0.43 μg (Supplementary Table S1). Therefore, we evaluated the performance of the WGA4 kit for the enrichment of recovered DNA, and it led to 3–1,330-fold amplification of the DNA (median = 37; *n* = 42), with ranging from 0.16 μg up to 8.25 μg (Supplementary Table S1). The WGA4 kit performs random fragmentation and amplification of genomic DNA and has been shown to increase DNA yield hundreds to million-fold (El-Heliebi et al., 2015; Ning et al., 2015), thus providing sufficient quantities of DNA for various NGS technologies (Ning et al., 2015; Ogunremi et al., 2020). Additionally, WGA4 in comparison to PicoPlex WGA kit for the detection of copy number variations in B-lymphocyte cell lines has been shown to produce a higher and more uniform yield and less GC-bias in amplification (Chen et al., 2018). However, the quality of DNA obtained after WGA4 amplification, as assessed with the A_260_/A_280_ ratio, was lower than the recommended range of 1.8–2.0 due to retention of salts, proteins, enzymes and other buffer components used in the amplification process. The DNA quality was significantly improved after the use of the GeneJet PCR clean up kit (Table 1), removing any inhibitors to the downstream MinION and Ion S5 sequencing procedures.

Currently, the detection and diagnosis of parasite infections rely on several laboratory methods, which are labor-intensive, time consuming and subjective, particularly microscopy (Ricciardi and Ndao, 2015). Biochemical assays often have low sensitivity and/or specificity, long turnaround time and limited scope (Momčilović et al., 2019; Ndao, 2009). In the past few decades there has been a focus on the development of molecular tools such as PCR, qPCR, multiplex and nested PCR for parasite detection and diagnostics (Shapiro et al., 2019). Although assays, such as multiplex real-time PCR, can simultaneously detect several parasite species, they are dependent upon specific molecular targets, which may not be readily available for all parasites (Momčilović et al., 2019). Metagenomic sequencing approaches, have successfully been used for the identification of bacterial pathogens more rapidly and precisely than traditional methods (Gu et al., 2019; Ogunremi et al., 2020; Wylezich et al., 2019). These approaches sequence the entire DNA content in a sample (Ogunremi et al., 2020), in contrast to targeted approaches that utilize singleplex or multiplex PCR reactions, thus substantially increasing the breadth of pathogens that can be detected (Pallen, 2014; Chiang and Dekker, 2020). There is very little work carried out to date on the metagenomic analysis of protozoan parasite contaminates in food. We are aware of a single, recent study where shotgun metagenomic sequencing was performed using the Illumina platform on three lettuce samples to enable microbial source tracking in a cryptosporidiosis outbreak in Sweden (Ahlinder et al., 2022). The analysis resulted in the detection of sequence reads identified as *C. parvum, C. hominis* and *C. muris* by the Centrifuge software and which mapped to *C. parvum* Iowa II strain reference genome (Accession number NC_006980). Very low frequencies of reads were obtained in the study and there did not appear to be a sufficient and reproducible discrimination between the different *Cryptosporidium* species.

We have employed the use of third generation whole genome sequencing using the nanopore technology for the detection of parasites from intentionally contaminated lettuce by sequencing metagenomic DNA obtained from the surface of the lettuce. The MinION is the smallest sequencing device currently on the market and thus the most portable: the dimensions are 10 cm × 3 cm × 2 cm and the weight is only 87 g. Nucleotide sequences are deduced from electrical current disruptions that occur when DNA passes through nanopores of the sequencing flow cell. This technology is orders of magnitude faster than other strategies that use sequence-by-synthesis methods and sequence data are available in real-time. In our study, the identification of parasites from the MinION sequenced data was carried out by simply submitting the reads to the CosmosID webserver. We chose CosmosID as our method of analysis because of our recent study wherein four bioinformatic approaches were compared for the identification of *Salmonella* from intentionally contaminated lettuce, and CosmosID was shown to produce superior results in identifying as few as one colony forming unit of *Salmonella* (Ogunremi et al., 2020). The CosmosID analysis relies on a *k-mer* algorithm screening of submitted reads against highly accurate and curated databases for bacteria, parasites, viruses and fungi. The identification of contaminants is usually obtained within a few minutes, depending upon the workload on the CosmosID server. In the current study, we detected as few as 2 oocysts, however consistent identification was achieved at 100 oocysts or greater (Table 1 and Supplementary Table S1). Paulos et al. (2019) evaluated four commercial multiplex real-time PCR assays for the detection of *Cryptosporidium hominis/parvum, Giardia duodenalis* and *Entamoeba histolytica*. The methods tested included: (1) Gastroenteritis/parasite test (Diagenode); (2) Ridagene parasitic stool test (R-Biopharm); (3) Allplex gastrointestinal parasite test (Seegene); and (4) FTD stool parasites (Fast Track) real-time PCR assay. The R-Biopharm method was shown to be the most sensitive assay at 87.5% diagnostic sensitivity for *C. hominis/parvum* and 78.7% for *G. duodenalis*. The detection limit in serially diluted samples positive for *Cryptosporidium* was 10^3^ oocysts, achieved with the R-Biopharm, which was two orders of magnitude more sensitive than other three assays (Paulos et al., 2019). Recently, Iqbal et al. (2019) developed an assay based on aptamer-coupled magnetic beads and aptasensors for the detection of *C. parvum* oocysts in drinking and recreational water, and demonstrated a detection limit of 50 oocysts, although the best sensitivity was achieved for samples spiked with 100–700 oocysts. However, the cross reactivity of this assay with other closely-related *Cryptosporidium* spp., such as *C. hominis* and *C. muris*, was not determined. Conversely, the MinION assay described in the current study successfully identified and differentiated *C. parvum, C. hominis* and *C. muris*, as well as *G. duodenalis* and *Toxoplasma gondii*, all from the same sample.

A novel multiplex PCR with a detection limit of 1–10 oocysts/cysts per gram of spinach (translating to 25–250 oocysts/cysts in 25 g of lettuce) for four different parasites was recently published (Shapiro et al., 2019). The assay was evaluated for the simultaneous detection of *C. parvum, G. duodenalis, Cyclospora cayetanensis*, and *T. gondii*. The DNA extraction method included both freeze-thaw cycles (4 min in liquid nitrogen and 4 min in boiling water) and proteinase K treatment at 56°C overnight. While this method seems to represent an advancement in parasite molecular diagnostics, the extraction still required an overnight incubation step. Furthermore, the specific identification of the parasites was only achieved after a two-step assay involving a multiplex PCR and either a nested PCR or a restriction fragment length polymorphism (RFLP) procedure (Shapiro et al., 2019). Furthermore, the assay was limited to the differentiation of only these four parasite species. In contrast, our lysis method is fast, simple and does not require freeze-thaw cycles or proteinase K treatment. Additionally, our parasite identification is based on mNGS, and this procedure can be used to identify and differentiate the different protozoan parasite species, as long as an appropriate and comprehensive reference database is available that includes the microbial contaminant. All the steps involved in our entire mNGS detection protocol including parasite lysis, DNA recovery, sequencing and bioinformatics analysis can be completed in just under 12 h, which is considerably faster than any other available parasite detection methods currently available. For some methods, the extraction procedure alone takes just as long when an overnight incubation is required. Furthermore, our MinION sequencing step is much faster, i.e., within a few hours, compared to the commonly used alternate platform, namely Illumina, which usually takes 2 days.

In this study, we also specifically evaluated the length of the run of the MinION sequencing procedure required to achieve reliable parasite identification. We developed a bioinformatic script that segregated MinION sequencing reads according to time of sequencing. In our time series experiments, we were able to generate sequence data for the successful identification and differentiation among various parasite species within 1.5 h for samples spiked with 1,000 oocysts of *C. parvum* per 25 g of lettuce. In contrast, other molecular methods such as PCR are time consuming and often require secondary methods such as nested PCR, amplicon sequencing or RFLP for identification, and target only a limited number of pathogens using specific primers or probes. Although very few mNGS methods have been reported for parasites (Wylezich et al., 2019), they have been shown to be very effective in detecting and identifying bacterial and viral species/strains in a short time under conditions relevant for diagnostic testing (Doan et al., 2017; Gu et al., 2019; Ogunremi et al., 2020). In the current study, selected MinION results were validated with an Ion S5 platform, capable of producing millions of reads with an average read length of 400 bases within 2.5 to 4.5 h. The Ion S5 has been consistently used for sequencing whole genomes, targeted gene panels, exomes, transcriptomes and metagenomes (Braukmann et al., 2019; Cifaldi et al., 2019; Mehrotra et al., 2017; Xue et al., 2018). There was a strong agreement in the results of the identification of parasites on lettuce using the MinION and Ion S5 sequencing platforms when spiked with a parasite dose of ≥10 oocysts. To the best of our knowledge, this is the first study showing rapid lysis coupled with quick parasite identification, yet our study had some limitations such as (1) we air dried the spiked lettuce for about 15 min, albeit with good aeration but this may not necessarily ensure optimal adherence of the parasites to plant cells. Hence, further studies testing different drying times are required to assess the effects of the drying time on parasite recovery; (2) Only a single sample of *T. gondii* was tested and other parasites such *C. muris* and *C. hominis* were not tested at low numbers. Further studies with more replicates at low numbers are required to fully validate our approach.

## 5 Conclusion

We have developed an approach that allows for the disruption of parasite cysts and oocysts within 3 min, followed by the amplification of DNA over 4.5 h to generate an adequate quantity and quality for next generation sequencing technologies. Based on mNGS using a MinION sequencer, we have developed a rapid, precise and objective approach that can simultaneously detect, identify and differentiate *C. parvum, C. hominis, C. muris, G. duodenalis* and *Toxoplasma gondii* on lettuce and be completed within 12 h. The method is sensitive and specific and has the potential to be used in diagnostic laboratories, in the field for food and environmental testing, and potentially for the subtyping of parasites for foodborne outbreak investigations and surveillance studies.

## Data Availability

The datasets presented in this study can be found in online repositories. The names of the repository/repositories and accession number(s) can be found below: https://www.ncbi.nlm.nih.gov/genbank/, PRJNA1077264.

## References

[B1] Ahlinder J. Svedberg A-. L. Nystedt A. Dryselius R. Jacobsson K. Hägglund M.. (2022). Use of metagenomic microbial source tracking to investigate the source of a foodborne outbreak of cryptosporidiosis. Food Waterborne Parasitol. 26:e00142. 10.1016/j.fawpar.2021.e00142 35024477 PMC8728467

[B2] Braukmann T. W. A. Ivanova N. V. Prosser S. W. J. Elbrecht V. Steinke D. Ratnasingham S.. (2019). Metabarcoding a diverse arthropod mock community. Mol. Ecol. Resour. 19, 711–727. 10.1111/1755-0998.13008 30779309 PMC6850013

[B3] Budu-Amoako E. Greenwood S. J. Dixon B. R. Barkema H. W. McClure J. T. (2011). Foodborne illness associated with *Cryptosporidium* and *Giardia* from livestock. J. Food Prot. 74, 1944–1955. 10.4315/0362-028X.JFP-11-107 22054199

[B4] Checkley W. White A. C. Jaganath D. Arrowood M. J. Chalmers R. M. Chen X-. M.. (2015). A review of the global burden, novel diagnostics, therapeutics, and vaccine targets for Cryptosporidium Lancet Infect. Dis. 15, 85–94. 10.1016/S1473-3099(14)70772-8 25278220 PMC4401121

[B5] Chen D. Zhen H. Qiu Y. Liu P. Zeng P. Xia J.. (2018). Comparison of single cell sequencing data between two whole genome amplification methods on two sequencing platforms. Sci. Rep. 8:4963. 10.1038/s41598-018-23325-2 29563514 PMC5862989

[B6] Chiang A. D. Dekker J. P. (2020). From the pipeline to the bedside: advances and challenges in clinical metagenomics. J. Infect. Dis. 221, S331–S340. 10.1093/infdis/jiz151 31538184 PMC7325616

[B7] Cifaldi C. Brigida I. Barzaghi F. Zoccolillo M. Ferradini V. Petricone D.. (2019). Targeted NGS platforms for genetic screening and gene discovery in primary immunodeficiencies. Front. Immunol. 10:316. 10.3389/fimmu.2019.00316 31031743 PMC6470723

[B8] Dixon B. Parrington L. Cook A. Pintar K. Pollari F. Kelton D.. (2011). The potential for zoonotic transmission of *Giardia duodenalis* and *Cryptosporidium* spp. from beef and dairy cattle in Ontario, Canada. Vet. Parasitol. 175, 20–26. 10.1016/j.vetpar.2010.09.032 20971563

[B9] Dixon B. Parrington L. Cook A. Pollari F. Farber J. (2013). Detection of *Cyclospora, Cryptosporidium*, and *Giardia* in ready-to-eat packaged leafy greens in Ontario, Canada. J. Food Prot. 76, 307–313. 10.4315/0362-028X.JFP-12-282 23433379

[B10] Doan T. Acharya N. R. Pinsky B. A. Sahoo M. K. Chow E. D. Banaei N.. (2017). Metagenomic DNA sequencing for the diagnosis of intraocular infections. Ophthalmology 124, 1247–1248. 10.1016/j.ophtha.2017.03.045 28526549 PMC5533302

[B11] El-Heliebi A. Chen S. Kroneis T. (2015). “Heat-induced fragmentation and adapter-assisted whole genome amplification using genomeplex^®^ single-cell whole genome amplification kit (WGA4), p101-109.” in Whole Genome Amplification: Methods and Protocols, T Kroneis (editor). New York: Springer. 26374312 10.1007/978-1-4939-2990-0_7

[B12] Forbes J. D. Knox N. C. Ronholm J. Pagotto F. Reimer A. (2017). Metagenomics: the next culture-independent game changer. Front. Microbiol. 8:1069. 10.3389/fmicb.2017.01069 28725217 PMC5495826

[B13] Gamble H. R. (2015). Trends in food production practices relative to foodborne parasites,” in Foodborne Parasites in the Food Supply Web, A. A. Gajadhar (editor). Oxford: Woodhead publishing. p. 11–2236744224

[B14] Gomez C. A. Sahoo M. K. Kahn G. Y. Zhong L. Montoya J. G. Pinsky B. A.. (2019). Dual-target, real-time PCR for the diagnosis of intraocular *Toxoplasma gondii* infections. Br. J. Ophthalmol. 103, 569–572. 10.1136/bjophthalmol-2018-313064 30636207 PMC6691874

[B15] Gu W. Miller S. Chiu C. Y. (2019). Clinical metagenomic next-generation sequencing for pathogen detection. Annu. Rev. Pathol. 14, 319–338. 10.1146/annurev-pathmechdis-012418-012751 30355154 PMC6345613

[B16] Hart M. L. Meyer A. Johnson P. J. Ericsson A. C. (2015). Comparative evaluation of DNA extraction methods from feces of multiple host species for downstream next-generation sequencing. PLoS ONE 10:e0143334. 10.1371/journal.pone.0143334 26599606 PMC4657925

[B17] Hawash Y. (2014). DNA extraction from protozoan oocysts/cysts in feces for diagnostic PCR. Korean J. Parasitol. 52, 263–271. 10.3347/kjp.2014.52.3.263 25031466 PMC4096637

[B18] Iqbal A. Goldfarb D. M. Slinger R. Dixon B. R. (2015). Prevalence and molecular characterization of *Cryptosporidium* spp. and *Giardia duodenalis* in diarrhoeic patients in the Qikiqtani region, Nunavut, Canada. Int. J. Circumpolar Health 74:27713. 10.3402/ijch.v74.27713 26095244 PMC4475686

[B19] Iqbal A. Liu J. Dixon B. Zargar B. Sattar S. A. (2019). Development and application of DNA-aptamer-coupled magnetic beads and aptasensors for the detection of *Cryptosporidium parvum* oocysts in drinking and recreational water resources. Can. J. Microbiol. 65, 851–857. 10.1139/cjm-2019-0153 31404505

[B20] Kelly R. P. Shelton A. O. Gallego R. (2019). Understanding PCR processes to draw meaningful conclusions from environmental DNA studies. Sci. Rep. 9:12133. 10.1038/s41598-019-48546-x 31431641 PMC6702206

[B21] Lalonde L. F. Gajadhar A. A. (2016). Detection of *Cyclospora cayetanensis, Cryptosporidium* spp., and *Toxoplasma gondii* on imported leafy green vegetables in Canadian survey. Food Waterborne Parasitol 2, 8–14. 10.1016/j.fawpar.2016.01.001

[B22] Mechan-Llontop M. E. Sharma P. Aguilera Flores M. Yang S. Pollock J. Tian L.. (2020). Strain-level identification of bacterial tomato pathogens directly from metagenomic sequences. Phytopathology 110, 768–779. 10.1094/PHYTO-09-19-0351-R 31829116

[B23] Mehrotra M. Duose D. Y. Singh R. R. Barkoh B. A. Manekia J. Harmon M. A.. (2017). Versatile ion S5XL sequencer for targeted next generation sequencing of solid tumors in a clinical laboratory. PLoS ONE 12:e0181968. 10.1371/journal.pone.0181968 28767674 PMC5540534

[B24] Momčilović S. Cantacessi C. Arsić-Arsenijević V. Otranto D. Tasić-Otašević S. (2019). Rapid diagnosis of parasitic diseases: current scenario and future needs. Clin. Microbiol. Infect. 25, 290–309. 10.1016/j.cmi.2018.04.028 29730224

[B25] Nair H. P. Vincent H. Bhat S. G. (2014). Evaluation of five in situ lysis protocols for PCR amenable metagenomic DNA from mangrove soils. Biotechnol Rep (Amsterdam, Netherlands) 4, 134–138. 10.1016/j.btre.2014.09.008 28626672 PMC5466134

[B26] Ndao M. (2009). Diagnosis of parasitic diseases: old and new approaches. Interdiscip. Perspect. Infect. Dis. 2009:278246. 10.1155/2009/278246 20069111 PMC2804041

[B27] Ning L. Li Z. Wang G. Hu W. Hou Q. Tong Y.. (2015). Quantitative assessment of single-cell whole genome amplification methods for detecting copy number variation using hippocampal neurons. Sci. Rep. 5:11415. 10.1038/srep11415 26091148 PMC4650676

[B28] Ogunremi D. Dupras A. A. Naushad S. Gao R. Omidi K. Marquez I. G.. (2020). A new whole genome culture-independent diagnostic test (WG-CIDT) for rapid detection of *Salmonella* in lettuce. Front. Microbiol. 10:00602. 10.3389/fmicb.2020.00602 32362880 PMC7181323

[B29] Pallen M. J. (2014). Diagnostic metagenomics: potential applications to bacterial, viral and parasitic infections. Parasitology 141, 1856–1862. 10.1017/S0031182014000134 24576467 PMC4255322

[B30] Paulos S. Saugar J. M. Lucio d. e. Fuentes A. Mateo I. Carmena M. D.. (2019). Comparative performance evaluation of four commercial multiplex real-time PCR assays for the detection of the diarrhoea-causing protozoa *Cryptosporidium hominis/parvum, Giardia duodenalis* and *Entamoeba histolytica*. PLoS ONE 14:e0215068. 10.1371/journal.pone.0215068 30958837 PMC6453453

[B31] Pomari E. Piubelli C. Perandin F. Bisoffi Z. (2019). Digital PCR: a new technology for diagnosis of parasitic infections. Clin. Microbiol. Infect. 25, 1510–1516. 10.1016/j.cmi.2019.06.009 31226445

[B32] Prystajecky N. Huck P. M. Schreier H. Isaac-Renton J. L. (2014). Assessment of *Giardia* and *Cryptosporidium* spp. as a microbial source tracking tool for surface water: application in a mixed-use watershed. Appl. Environ. Microbiol. 80, 2328–2336. 10.1128/AEM.02037-13 24463970 PMC3993175

[B33] Ricciardi A. Ndao M. (2015). Diagnosis of parasitic infections: what's going on? J. Biomol. Screen. 20, 6–21. 10.1177/1087057114548065 25170017

[B34] Robertson L. J. (2018). “Chapter four - parasites in food: from a neglected position to an emerging issue,”. in Advances in Food and Nutrition Research, vol 86, D. Rodríguez-Lázaro (editor). Cambridge: Academic Press. p. 71–113. 30077225 10.1016/bs.afnr.2018.04.003 PMC7129657

[B35] Robertson L. J. Torgerson P. R. van der Giessen J. (2018). Foodborne parasitic diseases in Europe: social cost-benefit analyses of interventions. Trends Parasitol. 34, 919–923. 10.1016/j.pt.2018.05.007 29921499

[B36] Ryan U. Hijjawi N. Feng Y. Xiao L. (2019). *Giardia*: an under-reported foodborne parasite. Int. J. Parasitol. 49, 1–11. 10.1016/j.ijpara.2018.07.003 30391227

[B37] Shapiro K. Kim M. Rajal V. B. Arrowood M. J. Packham A. Aguilar B.. (2019). Simultaneous detection of four protozoan parasites on leafy greens using a novel multiplex PCR assay. Food Microbiol. 84:103252. 10.1016/j.fm.2019.103252 31421749

[B38] Squire S. A. Ryan U. (2017). *Cryptosporidium* and *Giardia* in Africa: current and future challenges. Parasit. Vectors 10:195. 10.1186/s13071-017-2111-y 28427454 PMC5397716

[B39] Thivierge K. Iqbal A. Dixon B. Dion R. Levesque B. Cantin P.. (2016). *Cryptosporidium hominis* is a newly recognized pathogen in the arctic region of Nunavik, Canada: molecular characterization of an outbreak. PLoS Neglect Trop D 10:e0004534. 10.1371/journal.pntd.0004534 27058742 PMC4825996

[B40] Torgerson P. R. Devleesschauwer B. Praet N. Speybroeck N. Willingham A. L. Kasuga F.. (2015). World Health Organization estimates of the global and regional disease burden of 11 foodborne parasitic diseases, 2010: a data synthesis. PLoS Med. 12:e1001920. 10.1371/journal.pmed.1001920 26633705 PMC4668834

[B41] Vandeventer P. E. Weigel K. M. Salazar J. Erwin B. Irvine B. Doebler R.. (2011). Mechanical disruption of lysis-resistant bacterial cells by use of a miniature, low-power, disposable device. J. Clin. Microbiol. 49, 2533–2539. 10.1128/JCM.02171-10 21543569 PMC3147885

[B42] Vizon K. C. C. Battad Z. G. Castillo D. S. C. (2019). Contamination of food-borne parasites from green-leafy vegetables sold in public markets of San Jose City, Nueva Ecija, Philippines. J. Parasit. Dis. 43, 651–657. 10.1007/s12639-019-01144-0 31749537 PMC6841888

[B43] Wong Y-. P. Othman S. Lau Y-. L. Radu S. Chee H-. Y. (2018). Loop-mediated isothermal amplification (LAMP): a versatile technique for detection of micro-organisms. J. Appl. Microbiol. 124, 626–643. 10.1111/jam.13647 29165905 PMC7167136

[B44] Wylezich C. Belka A. Hanke D. Beer M. Blome S. Höper D.. (2019). Metagenomics for broad and improved parasite detection: a proof-of-concept study using swine faecal samples. Int. J. Parasitol. 49, 769–777. 10.1016/j.ijpara.2019.04.007 31361998

[B45] Xue J. Wu R. Pan Y. Wang S. Qu B. Qin Y.. (2018). Integrated massively parallel sequencing of 15 autosomal STRs and amelogenin using a simplified library preparation approach. Electrophoresis 39, 1466–1473. 10.1002/elps.201700429 29608791

