# Supplementary material for: Metagenomic detection of protozoan parasites on leafy greens aided by a rapid and efficient DNA extraction protocol
Source: Front Microbiol. 2025 Mar 14;16:1566579. doi: 10.3389/fmicb.2025.1566579 (PMC11949954; doi:10.3389/fmicb.2025.1566579)
Supplement: Supplementary file 2 [file Table_2.docx]

Supplementary Table S2 Timeline of metagenomic detection of *Cryptosporidium parvum*

extracted from lettuce

| Sample | Time from start of sequencing | Number of fastq reads | Detection of  *C. parvum* |
| --- | --- | --- | --- |
| Lettuce spiked with  1,000 oocysts of  *C. parvum* | 30 minutes | 370,356 | No |
|  | 60 minutes | 827,015 | No |
|  | 90 minutes | 1,296,583 | Yes |

The earliest detection of *C. parvum-*specific sequence reads in the metagenome of contaminated lettuce surface were determined using simulated time series experiments with the aid of nanoTimeSort v0.1 script (<https://github.com/duceppemo/nanoTimeSort>) performed on reads acquired at 30 min intervals We developed the script to simulate a time series study by segregating MinION output into 30 min of sequencing reads with sequential additions up to the end of the run at about 48 h. Once detected, parasite sequence were continually detected until the end of the experiment (data not shown).
