# Supplementary figures and images for: Metagenomic detection of protozoan parasites on leafy greens aided by a rapid and efficient DNA extraction protocol
Source: Front Microbiol. 2025 Mar 14;16:1566579. doi: 10.3389/fmicb.2025.1566579 (PMC11949954; doi:10.3389/fmicb.2025.1566579)

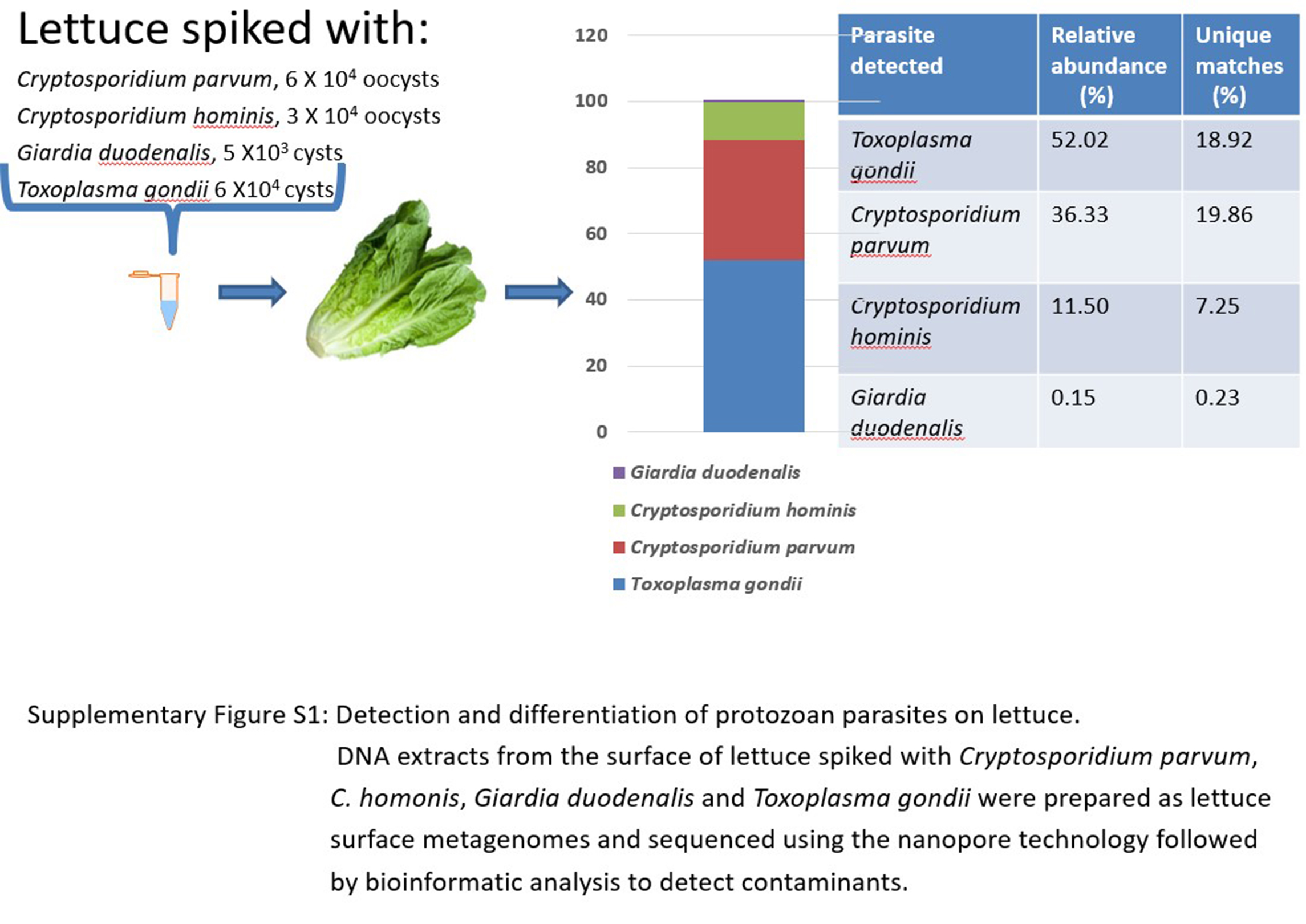

Supplement: Supplementary file 4 [file Image_1.jpg]
